# Hypoxic acclimatization training improves the resistance to motion sickness

**DOI:** 10.3389/fnins.2023.1216998

**Published:** 2023-12-06

**Authors:** Rui Wang, Yiquan Yan, Yateng Tie, Qi Zhang, Yikai Pan, Shuhan Li, Jieyi Fan, Chengfei Li, Xi Li, Yongchun Wang, Xiqing Sun, Tongmei Zhang, Xingcheng Zhao

**Affiliations:** ^1^Department of Aerospace Medical Training, School of Aerospace Medicine, Fourth Military Medical University, Xi’an, China; ^2^Department of Aerospace Physiology, School of Aerospace Medicine, Fourth Military Medical University, Xi’an, China; ^3^School of Aerospace Medicine, Fourth Military Medical University, Xi’an, China

**Keywords:** resistance to motion sickness, motion sickness, hypoxia acclimatization training, 3D roller training, spatial disorientation

## Abstract

**Objective:**

Vestibular provocation is one of the main causes of flight illusions, and its occurrence is closely related to the susceptibility of motion sickness (MS). However, existing training programs have limited effect in improving the resistance to motion sickness. In this study, we investigated the effects of hypoxia acclimatization training (HAT) on the resistance to motion sickness.

**Methods:**

Healthy military college students were identified as subjects according to the criteria. MS model was induced by a rotary chair. Experimental groups included control, HAT, 3D roller training (3DRT), and combined training.

**Results:**

The Graybiel scores were decreased in the HAT group and the 3DRT group and further decreased in the combined training group in MS induced by the rotary chair. Participants had a significant increase in blood pressure after the rotary chair test and a significant increase in the heart rate during the rotary chair test, but these changes disappeared in all three training groups. Additionally, LFn was increased, HFn was decreased, and LF/HF was increased accordingly during the rotary chair test in the control group, but the changes of these three parameters were completely opposite in the three training groups during the rotary chair test. Compared with the control group, the decreasing changes in pupillary contraction velocity (PCV) and pupillary minimum diameter (PMD) of the three training groups were smaller. In particular, the binocular PCV changes were further attenuated in the combined training group.

**Conclusion:**

Our research provides a possible candidate solution for training military pilots in the resistance to motion sickness.

## 1 Introduction

Spatial disorientation (SD) refers to an erroneous perception of position, altitude, or motion related to the plane of the earth’s surface, or a false perception relative to gravitational vertical. SD has become the most important cause of human error in flight accidents, accounting for more than 30% of all human errors in flight accidents (Sanchez-Tena et al., 2018; Kang et al., 2020; Meeks et al., 2022).

Vestibular provocation, one of the main causes of flight illusions, is associated with the functional stability of the vestibular organs (Demir and Aydin, 2021). Vestibular organs, also known as balance organs, are the main sensory organs of the human body’s balance system, which include two parts: semicircular canals (canalis semicircularis anterior, canalis semicircularis lateralis, and canalis semicircularis posterior) and otolith apparatus (saccule and utricle) (Onishi et al., 2022). When the human body carries out variable rotating speed movement, it will cause the reverse flow of lymphatic fluid in the semicircular canal, stimulate the receptors of vestibular nerve endings, and then produce motor sensation and somatic reflex (Holmes et al., 2003). In particular, motion sickness (MS) and vestibular dysfunction remain important causes of medical grounding (Haworth et al., 2021).

Many studies suggest that acclimation training is the most effective method of improving the resistance to MS. Training methods mainly include physical training, fixed roller, spiral ladder, and rotary chair (Wang et al., 2000; Clement et al., 2001; Li and Sun, 2022). However, these training methods are limited in the improvement of MS because these can only stimulate the MS from a single direction and cannot simultaneously stimulate the three pairs of semicircularis, the sacculus, and the utriculus. Our research team solved this problem well and achieved an obvious effect in improving MS by developing a three-dimensional (3D) rotated training system. Previous research found that the training effect of the 3D roller is significantly better than traditional rotary chair in improving the resistance to MS. After the 3D roller training, the qualification rate of the vestibular function of volunteers has increased by 44.4% (Wang et al., 2016; Sun et al., 2020; Pan et al., 2022). However, with the increasing performance of new fight aircraft, it has become an urgent problem to explore new training methods to further improve the resistance to MS based on the 3D roller.

Hypoxia is very closely related to the vestibular function. Hypoxic vertigo is caused by hypoxia which causes decreased functional stability of the vestibular function (Zhou L. et al., 2022). There have been reports that individuals may experience a combination of dizziness, loss of balance, and hearing loss during the period of acute hypoxia, especially at altitudes above 5,000 m (moderate-to-severe hypoxia) (Wagner et al., 2016; Li et al., 2018). Oxygen depletion causes vestibular asymmetry and apoptosis of vestibular neurons in patients with obstructive sleep apnea syndrome (OSAS) (Gallina et al., 2010). On the other hand, hypoxia preconditioning (HPC) and hypoxia acclimatization have good protective effects, and hypoxia acclimatization training has also been applied in the improvement of athletes’ competitive level and the treatment of some diseases (Bailey and Davies, 1997; Stellingwerff et al., 2019; Bruzzese et al., 2021; Liu et al., 2021). However, whether the hypoxia acclimatization training could improve the resistance to motion sickness remains unclear.

In this study, we investigated the effects of hypoxia acclimatization training on MS stability using a rotary chair-induced MS model. We also employed the 3D roller training group as a positive control group and explored whether hypoxia acclimatization training and 3D roller training synergistically improved resistance to MS. Assessment of vestibular function stability has always been difficult. In the present study, the Graybiel diagnostic criteria and vestibular function scale were employed to assess subjective improvement in MS induced by the rotary chair in each training group. We assessed the changes in cardiovascular modulation after MS stimulation of subjects in each training group by monitoring blood pressure and heart rate. We explored autonomic neuromodulatory changes after MS stimulation in these training groups by analyzing heart rate variability and pupillary light reflex.

## 2 Materials and methods

### 2.1 Participants

A total of 48 volunteers were selected from 52 healthy young men using the electric rotary chair. The mean age was 21.0 ± 1.3 years, and the mean body mass index was 22.6 ± 2.3 kg/m^2^. Participants were required to follow the inclusion criterion: (1) healthy young men, (2) no history of motion sickness or neurological diseases that may influence vestibular responses, (3) Graybiel scores≥5. Participants were excluded from (1) Graybiel scores<5 (*n* = 2) and (2) unfinished training tasks (*n* = 2). Four participants were interrupted during the test, and their data were excluded from the analysis. Qualified volunteers were forbidden to take any drug or related beverage that affected the function of the vestibular autonomic nervous system within 48 h before and during the trial, and they had normal rest and diet without any strenuous exercise. Forty-eight qualified volunteers were randomly assigned to four groups: no training group (control group), hypoxic acclimatization training group (HAT), 3D roller training group (3DRT), and combined training group with hypoxic acclimatization and 3D roller training group (HAT + 3DRT), and each group had twelve individuals. The content of this trial was approved by the Medical Ethics Committee of Fourth Military Medical University (KY20232110-F-1). Before the beginning of this research, all participants were fully aware of the content of this trial and signed the informed consent.

### 2.2 Rotary chair test

The electric rotary chair applied in this experiment was realized by using the uniaxial motion of a 3D roller designed by Fourth Military Medical University and manufactured by Beijing Airspeed Technology Co., Ltd. (Beijing, China), and its effect on MS and cardiovascular function was basically consistent with traditional rotary chair in the previous study (Demir and Aydin 2021). It can realize uniaxial clockwise or counterclockwise rotation, and the maximum angular velocity of the rotating chair can reach 180°/s. Before the test, the portable electronic sphygmomanometer was fixed on the left upper arm to measure blood pressure, and the ECG sensor connected to three electrode pieces was strapped to the chest. The first test data were pupillary light reflex using an automatic pupilometer. After the subjects sat upright on the chair and fastened all seat belts in the straight position, the blood pressure was measured, and then an ECG sensor was turned on and recorded for 5 min before rotation. Then, the angle rotation speed of the rotary chair was set at 180°/s for 90 s. During rotation, each subject was asked to swing their head from 30° leftward to 30° rightward followed by the beat (once every 2 s) using a metronome. Meanwhile, heart rate and electrocardiogram were monitored in real time through computer software. If the heart rate changed too fast or the electrocardiogram was abnormal during the monitoring process, we would ask how the subject was feeling and choose whether to terminate the rotation in time in order to prevent the occurrence of risk. After the end of the rotation, the blood pressure data and the pupillary light reflex were immediately detected again. Importantly, these two kinds of data were prone to fluctuation after the subject recovers, so it was necessary to measure these two values in seconds. Next, each subject was required to fill out the Graybiel diagnostic criteria and the vestibular function scale questionnaires. In these two questionnaires, the subjects used different grades to report their symptom of MS. The ECG sensor was removed approximately 5 min after the rotary chair test.

### 2.3 Hypoxic acclimatization training

This study developed a consecutive 5 days hypoxia pre-adaptation training with 1 h daily training. The hypobaric chamber in this trial was designed by the Fourth Military Medical University and manufactured by Yantai Hongyuan Oxygen Industrial Co., Ltd. (Yantai, Shandong, China). Before training, we explained the whole training procedure and requirements to trainees and then led them into the cabin. When the simulated altitude of the hypobaric chamber started to rise, we reminded the trainees that they were at the rising stage. In this training, the hypobaric chamber rose to an altitude of 3,000 m (equal to 14.4% oxygen at sea level) from 0 m at a constant speed of 15 m/s. After maintaining an altitude of 3,000 m for 1 h, it descended back to 0 m at a constant speed of 5 m/s. When the simulated altitude of the hypobaric chamber started to drop, we reminded participants that they were in the descending stage. The main reason for the different rates between ascent and descent is that the Eustachian tube is a one-way valve that opens automatically when the external pressure is low. During the descent process with high external pressure, the Eustachian tube is required to be opened by swallowing and chewing or by squeezing the nose, so that the descent process is at a lower speed.

### 2.4 3D roller training

**Figure 1:**
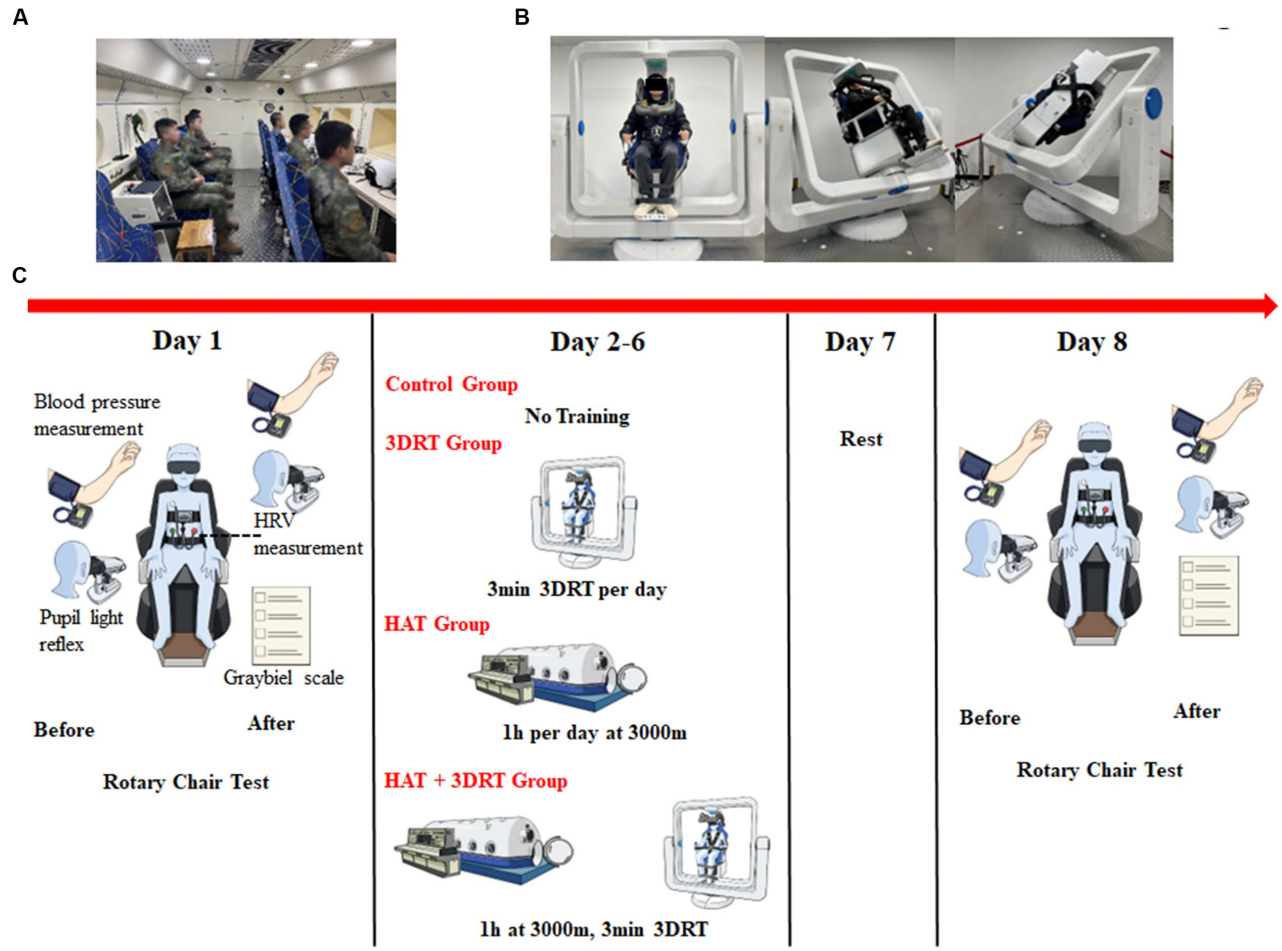
Photographs of hypoxia acclimatization training and 3D roller training and schematic diagram of the experimental procedure. **(A)** A representative photograph of participants undergoing hypoxia acclimatization training in the hypobaric chamber. **(B)** Three representative photographs of a participant undergoing vestibular function training by a 3D roller. **(C)** Schematic diagram of the experimental procedure.

The innovative electric 3D roller applied in this trial was designed and developed by the Air Force Medical University and manufactured by the Beijing Airspeed Technology Co., LTD. During operation, the maximum rotational angular velocity of the inner frame, middle frame, and outer frame is 180°/s, and the maximum rotational angular acceleration can reach 60°/s^2^. It can achieve multi-axial stimulation by setting rotational speed and angular acceleration of three frames, which triggers a series of MS. First, we assisted each participant to sit upright on the chair, fasten all seat belts, keep head close to the back of the seat, hold the handle, step on the pedal, and close eyes during the whole rotation process. The chair was mounted in a gimbal so that the participant was rotated as a consequence of the 3D roller motion (Figure 1B). Based on previous research experience (Pan et al., 2022), this test was updated to a consecutive 5-day progressive training program, respectively, setting different speeds of inner frame (60°/s, 70°/s, 80°/s, 80°/s, and 90°/s), middle frame (60°/s, 70°/s, 70°/s, 80°/s, and 80°/s), and outer frame (60°/s, 60°/s, 60°/s, 60°/s, and 60°/s) on each day. Meanwhile, the angular acceleration of three frames was 10°/s^2^ all the time, and the training duration was 3 min per day. Additionally, the HAT + 3DRT group was required to complete the above two training contents for HAT and then 3DRT on each day for 5 consecutive days.

### 2.5 Vestibular functional stability assessment and grading

At the end of each rotary chair test, the severity of motion sickness was evaluated by two questionnaires based on the “Graybiel diagnostic criteria” (Table 1) (Graybiel et al., 1968) and the “vestibular function scale” (Table 2) (Pan et al., 2022). Specifically, Graybiel diagnostic criteria were divided into seven categories with different symptom intensities, and each participant could select only one level of intensity per category or no selection if the symptom was not felt. Finally, the Graybiel score was calculated by adding the values of seven categories including nausea, skin color, cold sweating, increased salivation, drowsiness, pain, and some symptoms of the central nervous system such as headache and dizziness for each participant. In addition, motion sickness after the rotary chair in the vestibular function scale was divided into four degrees (0, I, II, and III) from no adverse reaction to severe symptoms. Each individual only chose one degree according to rotating feeling, and each choosing degree represented corresponding scores (0, 1, 2, and 3).

**Table 1:** Graybiel diagnostic criteria.

Category	Pathognomonic 16 points	Major 8 points	Minor 4 points	Minima 2 points	AQS 1 point
Nausea	Vomiting or retching	Nausea+ II, III	Nausea I	Epigastric discomfort	Epigastric awareness
Skin color		Pallor III	Pallor II	Pallor I	Flushing
Cold sweating		III	II	I	
Increased salivation		III	II	I	
Drowsiness			II	I	
Pain					Headache
Central nervous system					Dizziness: Eyes closed ≥II Eyes open III

AQS, additional qualifying symptoms; III, severe or marked; II, moderate, I, slight.

**Table 2:** Vestibular function scale.

Grading	Symptom
0	No adverse reaction
I	Minor dizziness, nausea, minor sweating, minor pallor, uncomfortable stomach
II	Dizziness, nausea, sweating, pallor
III	Obvious dizziness, headache, nausea, major sweating, pallor, vomiting or retching

### 2.6 Blood pressure measurement and heart rate variability data acquisition

A portable electronic sphygmomanometer J760 (Beijing Omron Medical Equipment Co., Ltd., Beijing, China) was applied to measure systolic blood pressure, diastolic blood pressure, and pulse number before and after the rotary chair test. The KF2 ECG sensor (Beijing Herserige Technology Co., Ltd., Beijing, China) was used to record heart rate, electrocardiogram, and respiratory rate during the rotary chair test, and heart rate variability (HRV) was analyzed using Damics 2021 software after the test. In the process of analysis, we selected the 90 s segment before, during, and after the rotary chair test. Indeed, although 90 s is relatively short for the analysis of heart rate variability, we selected 90 s basically for the following two reasons: one because the rotation process was a fixed segment of 90 s, and second, to maintain the same length of the statistics before, during, and after the rotation. The other important consideration was that cardiovascular changes are relatively temporary. To better reflect the instantaneous change trend, we selected the 90 s immediately after the end of the rotating chair test. In addition, both the analysis of heart rate and the respiration rate used the averaged value over each 90 s segment. HRV was analyzed in this trial using the frequency domain algorithm, and the tachogram was analyzed using the fast Fourier transform (FFT) parameter model method. Frequency domain analysis used NN interval discrete event series (DES) for analysis. HRV was also analyzed including normalized low frequency (LFn), normalized low frequency (HFn), and low frequency/high frequency (LF/HF). The sample rate of HRV is 200 Hz.

### 2.7 Pupillary light reflex

An automatic pupilometer (Suzhou Guoke Vision Medical Technology Co., Ltd., Suzhou, Jiangsu, China) was used to quantitatively measure the pupillary changes of the participant before and after the rotary chair test. This machine in this study measured four parameters such as pupil initial diameter (PID), pupil minimum diameter (PMD), pupil contraction velocity (PCV), and pupil contraction latency (PCL). PID and PMD referred to the pupil size before light stimulation, which reflected the balance relationship between the dilator major muscle (sympathetic nerve) and the globular muscle (parasympathetic nerve). Additionally, PCV indicated the maximum change rate of the pupil in response to light and PCL meant the time interval between the initial light stimulation and the onset of pupillary response. These two parameters mainly reflected the function of the parasympathetic nerve.

### 2.8 Statistical analysis

Plotting of statistics and statistical analyses were performed in GraphPad Prism 8.3.0. Comparisons between the groups were undertaken using the two-way ANOVA. The results were expressed as the mean ± standard deviation (SD) from 12 subjects per training group. A *p*-value of <0.05 was considered statistically significant.

## 3 Results

### 3.1 Hypoxia acclimatization and 3D roller training reduce Gabriel scores induced by a rotary chair

A Coriolis acceleration rotary chair is the main tool for vestibular functional stability training at present, and it can also induce the occurrence of motion sickness when combined with the head-swing movement of the participants (Zhao et al., 2022). In this study, hypoxia acclimatization training was carried out in the hypobaric chamber (Figure 1A). The 3D roller is a device independently developed by our research group. It can stimulate and acclimatize the three pairs of semicircular canals in the body by rotating in the *X*, *Y*, and *Z* axes, thereby improving the susceptibility of motion sickness (Figure 1B). The overall schematic of the experiment is shown in Figure 1C.

**Figure 2:**
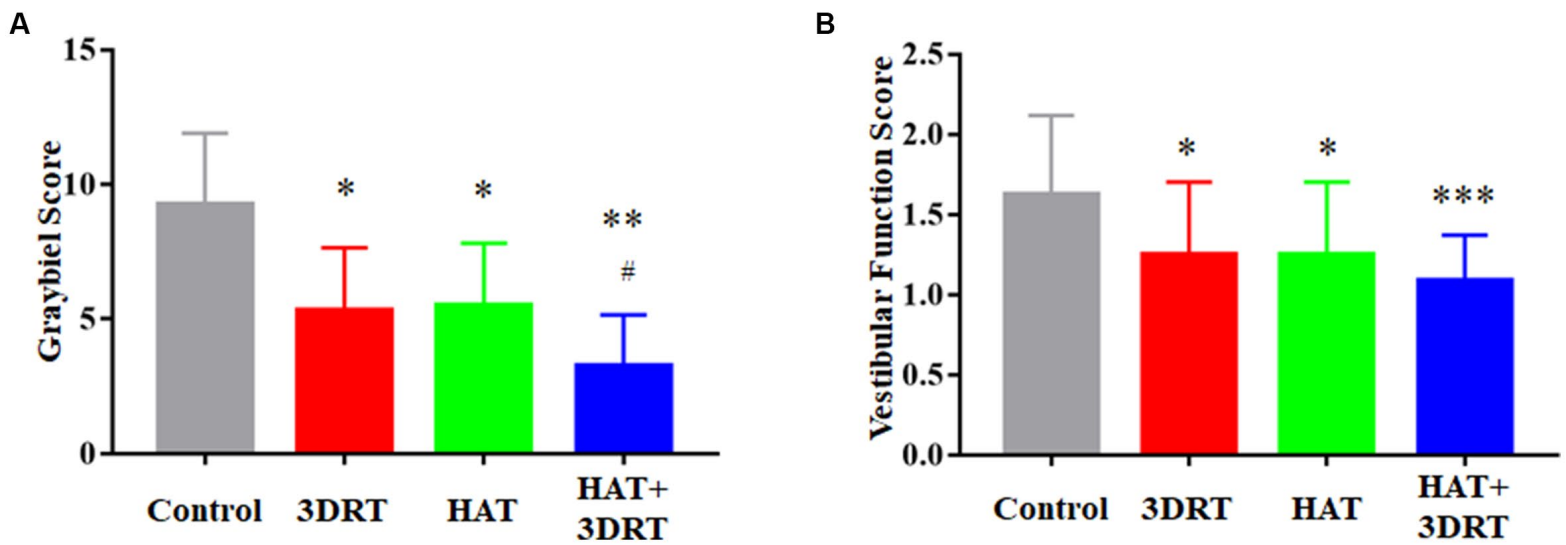
Hypoxia acclimatization training reduced the score of Graybiel and vestibular function scale induced by the rotary chair. **(A)** Graybiel score of the control group, 3DRT group, HAT group, and HAT + 3DRT group. **(B)** Vestibular function score of the control group, 3DRT group, HAT group, and HAT + 3DRT group. ^*^p < 0.05 compared with the control group. ^**^p < 0.01 and ^***^p < 0.001 compared with the control group. ^#^*p* < 0.05 compared with the HAT group.

The Graybiel scores were virtually unchanged in the control group in two rotary chair tests, indicating that the rotary chair induced MS successfully. After 1 week of training, the Graybiel score of the 3D roller training group decreased significantly, which was 5.33 ± 0.67 (*p* < 0.01 compared with before training 8.42 ± 0.875) points. There was also a significant reduction in the Graybiel score of 5.50 ± 0.73 (*p* < 0.01 compared with before training 9.20 ± 0.89) in the hypoxia training group, which was similar to that of the 3D roller training group. The Graybiel score was further reduced to 3.25 ± 0.67 (*p* < 0.01 compared with before training 7.50 ± 0.52) for the combined 3D roller and hypoxia acclimatization training group (Table 3 and Figure 2A).

**Table 3:** Comparison of Graybiel’s score before and after training by the rotary chair test.

	Control		3DRT		HAT		HAT + 3DRT	
	Before training	After training	Before training	After training	Before training	After training	Before training	After training
Gtaybiel’s score	9.160 ± 0.44	9.240 ± 0.53	8.42 ± 0.875^a^	5.33 ± 0.67^a^	9.20 ± 0.89^b^	5.50 ± 0.73^b^	7.50 ± 0.52^c^	3.25 ± 0.67^c^

^a^*p* < 0.01, ^b^*p* < 0.01, and ^c^*p* < 0.01.

Chinese military pilots use the vestibular function scale more often in vestibular function training and assessment, so we also performed statistical analyses of the subjects’ scores in the vestibular function scale accordingly. The score of the control group in the vestibular function scale was 1.63 ± 0.09 after the 90 s rotation of the rotary chair. After 1 week of training, the vestibular function classification score of the 3D roller training group decreased significantly, which was 1.25 ± 0.13 points (*p* < 0.05 compared with control). There was also a significant reduction in the vestibular function score of 1.25 ± 0.13 (*p* < 0.05 compared with control) in the hypoxia training group. The vestibular function classification score was 1.08 ± 0.08 in the combined 3D roller and hypoxia acclimatization training group (Figure 2B). Although there was no synergistic effect in vestibular function scores in the combined training group compared with the hypoxia training group or 3D roller training group, there was a further downward trend in the vestibular function scores in the combined training group. These results suggested that hypoxia acclimatization training could reduce the scores of Graybiel and the vestibular function scale induced by the rotary chair.

### 3.2 Hypoxia acclimatization training reduces the change in blood pressure and heart rate variability induced by a rotary chair

**Figure 3:**
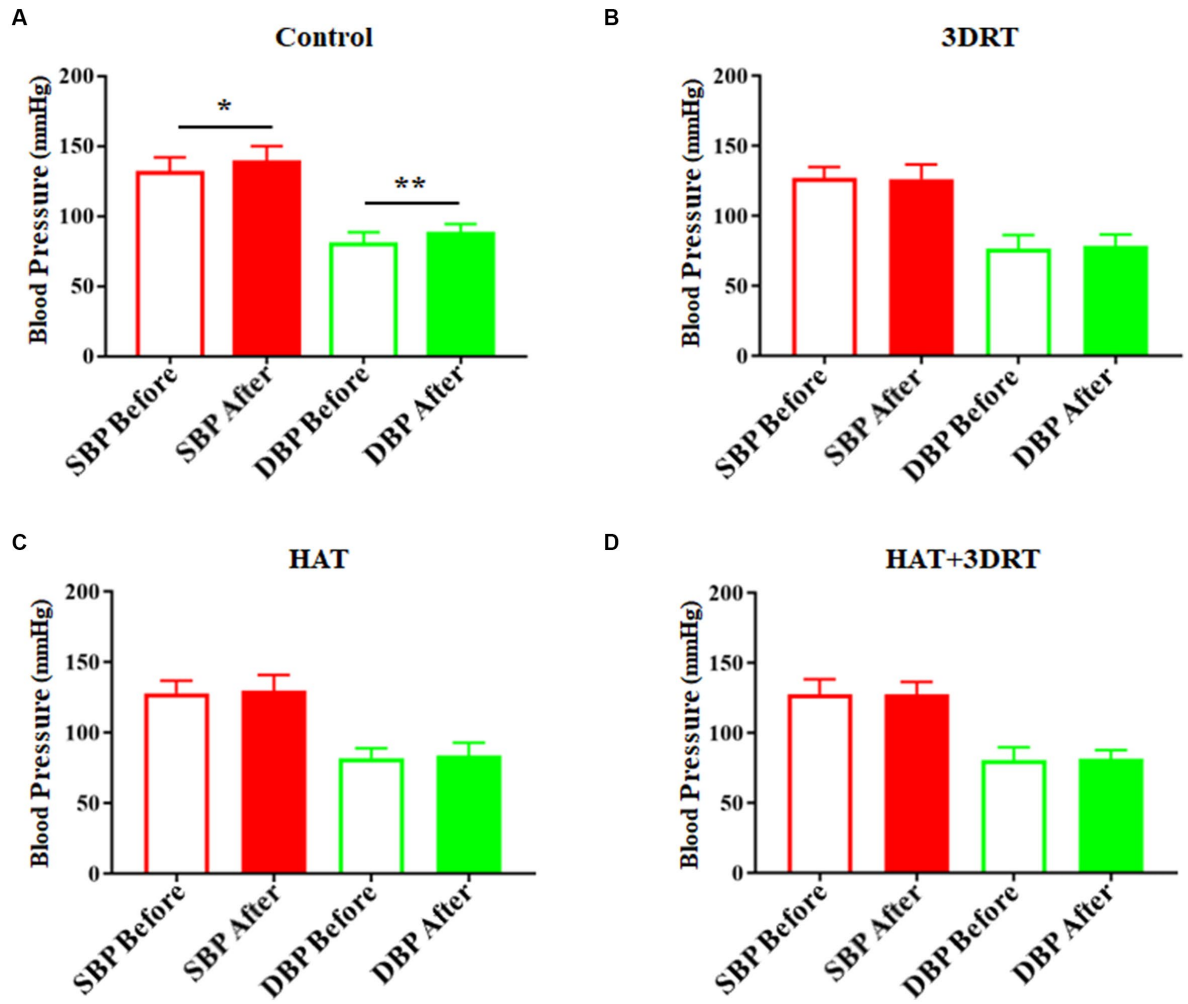
Hypoxia acclimatization training decreased the elevated blood pressure induced by the rotary chair. **(A)** Systolic and diastolic blood pressure of participants before and after the rotary chair test in the control group. **(B)** Systolic and diastolic blood pressure of participants before and after the rotary chair test in the 3DRT group. **(C)** Systolic and diastolic blood pressure of participants before and after the rotary chair test in the HAT group. **(D)** Systolic and diastolic blood pressure of participants before and after the rotary chair test in the HAT + 3DRT group. ^*^p < 0.05 and ^**^p < 0.01.

In Figure 3, the systolic blood pressure and the diastolic blood pressure were 131.10 ± 2.39 mmHg and 79.71 ± 2.15 mmHg, respectively in the control group before the rotary chair test. After the rotary chair test, the participants’ systolic blood pressure and diastolic blood pressure increased to 138.40 ± 2.55 (*p* < 0.05 compared with before) mmHg and 87.35 ± 1.71 mmHg (*p* < 0.01 compared with before), respectively, which was a significant increase (Figure 3). In the 3D roller training group, the systolic blood pressure and the diastolic blood pressure were 126.70 ± 2.31 mmHg and 76.33 ± 2.83 mmHg before the rotary chair test and 126.00 ± 3.02 mmHg and 78.25 ± 2.34 mmHg after the rotary chair test. In the hypoxia acclimatization training group, the systolic blood pressure and the diastolic blood pressure were 127.60 ± 2.64 mmHg and 81.64 ± 2.07 mmHg before the rotary chair test and 129.50 ± 3.38 mmHg and 83.18 ± 2.78 mmHg after the rotary chair test. In the combined 3D roller and hypoxia acclimatization training group, the systolic blood pressure and the diastolic blood pressure were 127.30 ± 3.09 mmHg and 80.36 ± 2.76 mmHg before rotary chair detection and 127.40 ± 2.56 mmHg and 81.45 ± 1.83 mmHg after rotary chair detection. These results indicate that similar to the 3D roller training group, participants in hypoxia acclimatization training had no significant changes in either systolic or diastolic blood pressure after the rotary chair testing.

**Figure 4:**
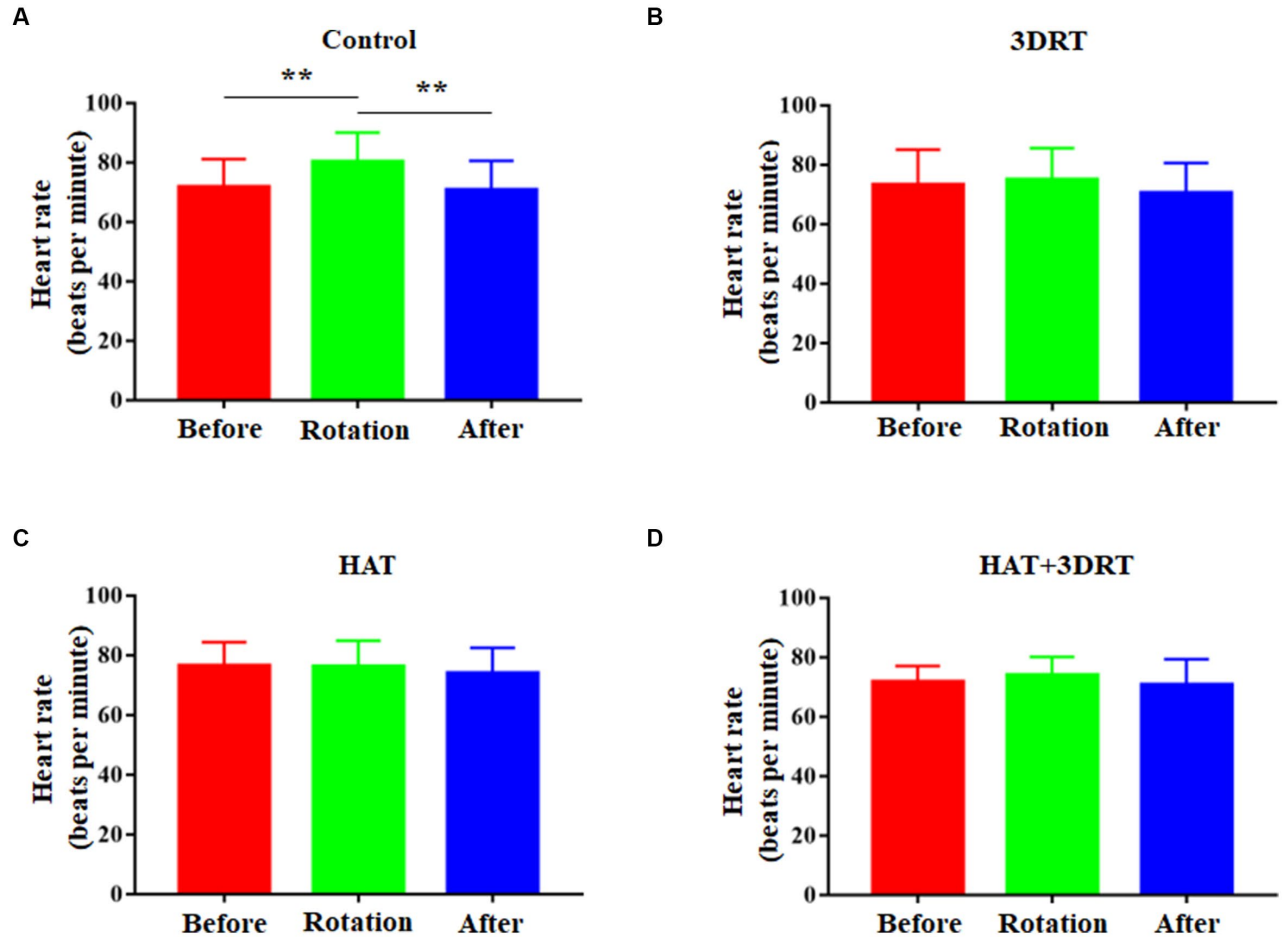
Hypoxia acclimatization training decreased the elevated heart rate induced by the rotary chair. **(A)** Heart rate of participants before, during, and after the rotary chair test in the control group. **(B)** Heart rate of participants before, during, and after the rotary chair test in the 3DRT group. **(C)** Heart rate of participants before, during, and after the rotary chair test in the HAT group. **(D)** Heart rate of participants before, during, and after the rotary chair test in the HAT + 3DRT group. ^**^p < 0.01.

In the control group, the heart rates increased from71.73 ± 1.99 to 80.27 ± 2.08 in the rotary chair test and then dropped to 70.70 ± 2.10 after the rotary chair test (Figure 4A). These results indicate that participants have a significant increase in the heart rate during the rotary chair test, but this change disappeared in all three training groups. In the 3D roller training group, the heart rates were 73.23 ± 3.75, 74.86 ± 3.42, and 70.35 ± 3.25 before, during, and after the rotary chair test, respectively (Figure 4B). In the hypoxia acclimatization training group, the heart rates were 76.37 ± 2.43, 76.21 ± 2.88, and 73.95 ± 2.82 before, during, and after the rotary chair test, respectively (Figure 4C). In the combined 3D roller and hypoxia acclimatization training group, the heart rates were 71.59 ± 1.81, 73.99 ± 2.03, and 70.64 ± 2.91 before, during, and after the rotary chair test, respectively (Figure 4D). All these results suggested that hypoxia acclimatization training reduces the changes of blood pressure and heart rate induced by the rotary chair. Hypoxia training improves cardiovascular regulation in participants in motion sickness induced by the rotary chair.

### 3.3 Hypoxia acclimatization training improved MS-induced autonomic response of HRV

**Figure 5:**
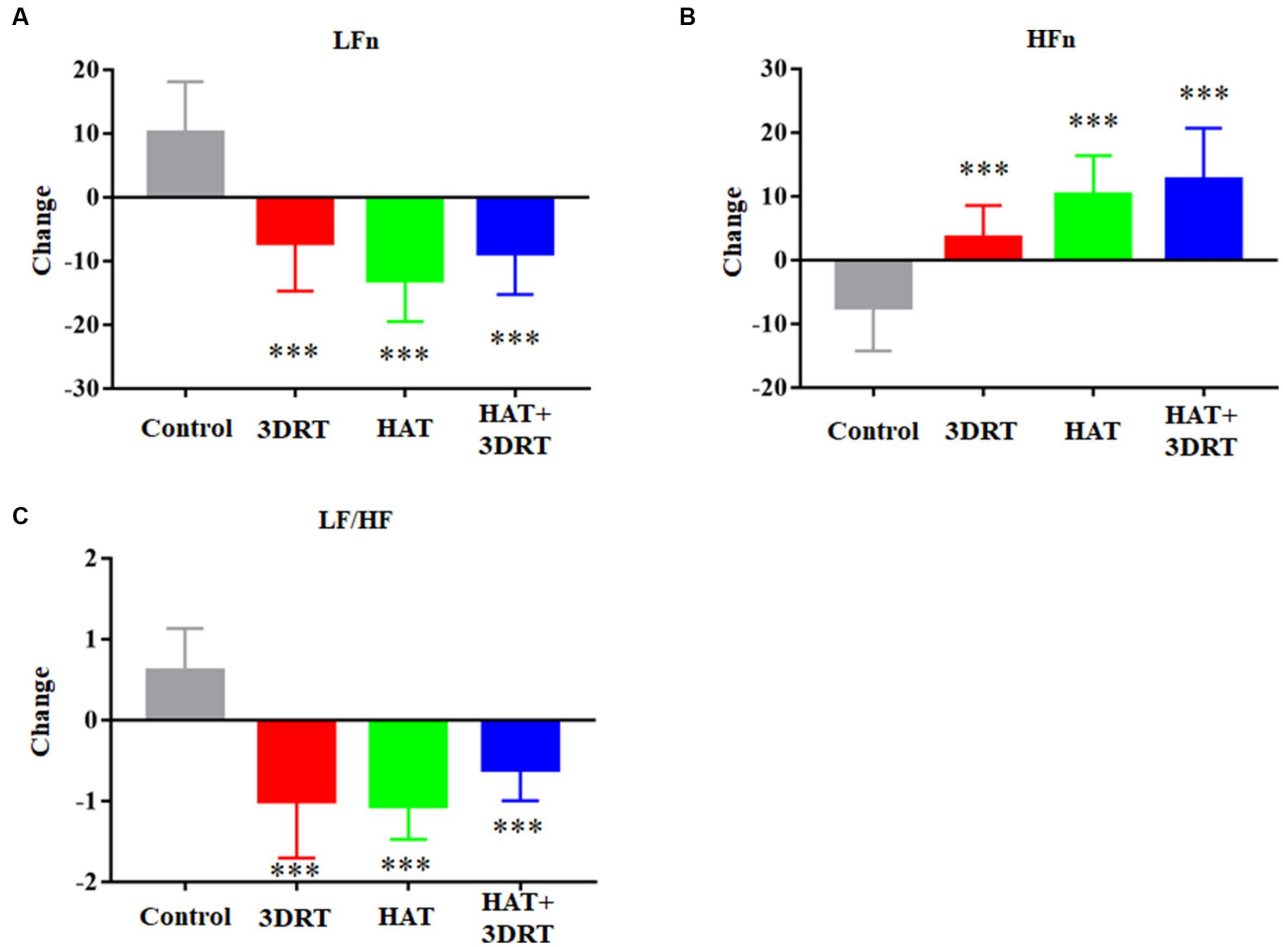
Hypoxia acclimatization training improved MS-induced autonomic response of HRV. **(A)** Changes of LFn in participants in the control, 3DRT, HAT, and HAT + 3DRT groups after rotary chair testing. **(B)** Changes of HFn in participants in the control, 3DRT, HAT, and HAT + 3DRT groups after rotary chair testing. **(C)** Changes of LF/HF in participants in the control, 3DRT, HAT, and HAT + 3DRT groups after rotary chair testing. ^***^p < 0.001 compared with the control group.

It is reported that the symptoms of MS are associated with autonomic nervous dysfunction. In our study, HRV signals were recorded by an ECG sensor during the rotary chair test. LF reflects sympathetic and vagal activity, HF reflects vagal activity, and LF/HF is a sensitive indicator of sympathetic and vagal tone balance. LFn and HFn are normalized LF and HF. In the control group, the LFn increased from 53.31 ± 11.17 to 63.38 ± 6.96, and the HFn decreased from 39.23 ± 13.39 to 31.80 ± 11.68 in the rotary chair test. In the 3D roller training group, the LFn decreased from 62.86 ± 11.43 to 60.61 ± 9.91 and the HFn increased from 21.87 ± 3.52 to 24.40 ± 4.94 in the rotary chair test. In the hypoxia acclimatization training group, the LFn decreased from 62.28 ± 13.05 to 55.55 ± 7.08, and the HFn increased from 27.97 ± 10.63 to 33.76 ± 9.04 in the rotary chair test. In the combined 3D roller and hypoxia acclimatization training group, the LFn decreased from 60.32 ± 12.25 to 51.59 ± 16.58, and the HFn increased from 30.18 ± 10.75 to 44.25 ± 20.28 in the rotary chair test (Table 4). We found LFn was increased significantly, HFn was decreased significantly, and LF/HF was increased accordingly during the rotary chair test, but in the three training groups, LFn was decreased significantly, HFn was increased significantly, and LF/HF was decreased accordingly during the rotary chair test (Figure 5). These results suggested that sympathetic activity was increased, and vagal activity was decreased in rotary chair-induced MS and hypoxia acclimatization training improved MS-induced autonomic response of HRV.

**Table 4:** Comparison of HRV before and in the rotary chair test.

	LFn		HFn		Total power (ms^2^)	
	Before rotary chair test	In rotary chair test	Before rotary chair test	In rotary chair test	Before rotary chair test	In rotary chair test
Control	53.313 ± 11.166^a^	63.377 ± 6.964^a^	39.230 ± 13.391	31.801 ± 11.678	6613.726 ± 4201.218	10698.528 ± 12349.112
3DRT	62.862 ± 11.426	60.607 ± 9.907	21.872 ± 3.518	24.397 ± 4.936	4041.336 ± 2351.087	6588.502 ± 4150.752
HAT	62.279 ± 13.052	55.546 ± 7.078	27.974 ± 10.632	33.764 ± 9.037	4163.355 ± 2080.506	3924.472 ± 2526.476
HAT + 3DRT	60.320 ± 12.251	51.588 ± 16.578	30.179 ± 10.750	44.251 ± 20.278	5525.023 ± 4246.802	5758.433 ± 4184.774

^a^*p* < 0.05.

### 3.4 Hypoxia acclimatization training improved pupillary light reflex measures

**Figure 6:**
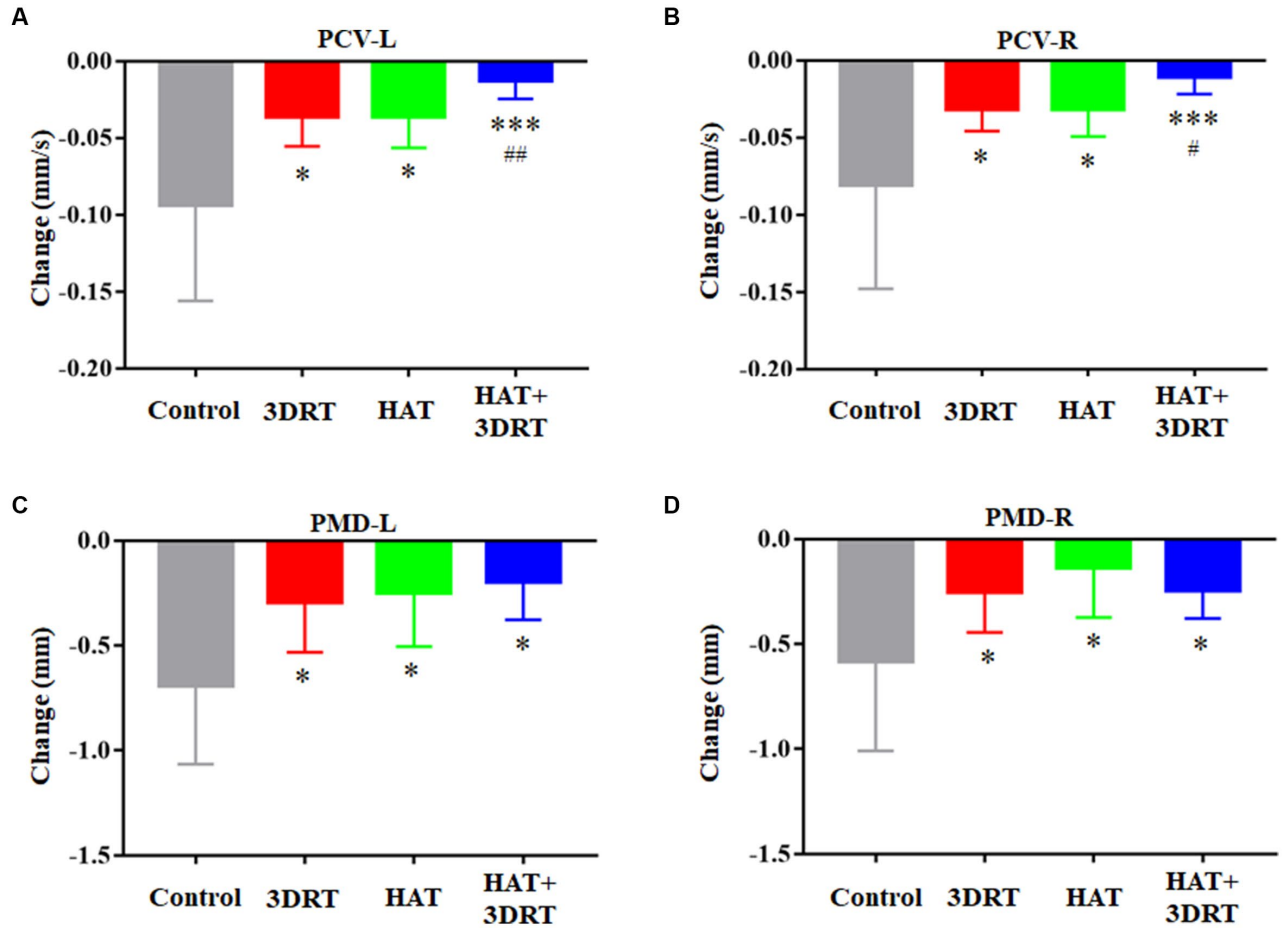
Hypoxia acclimatization training improved MS-induced autonomic response of PLR. **(A)** Changes of PCV of the left eye in participants in the control, 3DRT, HAT, and HAT + 3DRT groups after rotary chair testing. **(B)** Changes of PCV of the right eye in participants in the control, 3DRT, HAT, and HAT + 3DRT groups after rotary chair testing. **(C)** Changes of PMD of the left eye in participants in the control, 3DRT, HAT, and HAT + 3DRT groups after rotary chair testing. **(D)** Changes of PMD of the right eye in participants in the control, 3DRT, HAT, and HAT + 3DRT groups after rotary chair testing. ^*^p < 0.05 compared with the control group. ^*^p < 0.05, ^**^p < 0.01, and ^***^p < 0.001 compared with the control group. ^#^*p* < 0.05 and ^##^*p* < 0.01 compared with the HAT group.

To investigate the modulation of hypoxia acclimatization training on MS-induced autonomic response, we also tested the pupillary light reflex of the participants. Left eye and right eye pupillary contraction velocity (PCV) decreased by 0.094 ± 0.018 mm/s and 0.081 ± 0.017 mm/s, respectively, after the induction of motion sickness induced by the rotary chair. Left eye and right eye pupillary minimum diameter (PMD) decreased by 0.69 ± 0.09 mm and 0.58 ± 0.09 mm, respectively, after the induction of motor sickness by rotary chair. These results suggested that MS induced decreased autonomic neuromodulatory capacity. In the 3D roller training group, left eye and right eye PCV decreased by 0.036 ± 0.006 mm/s and 0.032 ± 0.005 mm/s, and left eye and right eye PMD decreased by 0.29 ± 0.11 mm and 0.25 ± 0.07 mm, respectively after induction of motor sickness by the rotary chair. In the hypoxia acclimatization training group, left eye and right eye PCV decreased by 0.036 ± 0.007 mm/s and 0.032 ± 0.006 mm/s, and left eye and right eye PMD decreased by 0.25 ± 0.10 mm and 0.14 ± 0.10 mm after induction of motor sickness by rotary chair, respectively. In the combined 3D roller and hypoxia acclimatization training group, left eye and right eye PCV decreased by 0.012 ± 0.04 mm/s and 0.011 ± 0.005 mm/s, and left eye and right eye PMD decreased by 0.20 ± 0.10 mm and 0.24 ± 0.08 mm after induction of motor sickness by the rotary chair, respectively (Figure 6). These results indicated that the change of PCV and PMD decreased after hypoxia acclimatization training in participants with MS induced by the rotary chair. Hypoxia acclimatization training improves MS-induced decrease in autonomic neuromodulatory capacity.

## 4 Discussion

In this study, we aimed to investigate the effect of hypoxia acclimatization training on MS. We also investigated the effect of 3D roller training on MS for two reasons. First, 3D roller training is a successful rehabilitation process and has been proven in our team’s previous research that it can affect MS susceptibility and the cardiovascular system (Sun et al., 2020; Pan et al., 2022). In this study, we used the 3D roller training as a positive control for the improvement of resistance to MS in participants using the rotary chair. Second, given that hypoxia acclimatization training improved resistance to MS, we investigated whether hypoxia acclimatization training and 3D roller training had synergistic effects on improved resistance to MS. Fortunately, some results showed that the hypoxic acclimation training and 3D roller training do have a synergistic effect on improving resistance to MS in participants (Figures 2A, 6A,B).

In the pre-experiment, we compared the results of 4,000 m 1 h and 3,000 m 1 h for hypoxia acclimatization and found that the results of 3,000 m 1 h were better. If the height continues to increase, there is a risk of altitude decompression sickness for the subjects. Therefore, the final parameter selection was 3,000 m 1 h for 5 days. Similar schemes have been applied in mice. Adamovich et al. used 14% oxygen (approximately 3,000 m) for 2 h in the hypoxic acclimatization for mice, but only once had a good hypoxic preconditioning effect (Adamovich et al., 2017).

The MS stimulation changes caused by the rotary chair after the hypoxia acclimatization training and 3D roller training were both lower compared with control, irrespective of the scores of subjective feelings or cardiovascular data. These results indicated that the resistance to motion sickness has been improved to some extent by the two different training methods, and their improvements were similar without significant differences. On the one hand, the advantages of the 3D roller training are more obvious. The biggest benefit of 3D roller training is more convenient with less training time. In addition, it could give military pilots a three-dimensional motion experience similar to flying a fighter plane. On the other hand, there are some superiorities of the hypoxia acclimatization training. First, some participants experienced dizziness and nausea during the 3D roller training, and the hypoxic acclimatization training could improve the MS without causing these symptoms. Second, the hypoxic acclimatization training not only improves the susceptibility to motion sickness but also improves hypoxic tolerance, which is very advantageous for military pilots.

Although there is no accurate method to quickly assess the resistance of motion sickness, based on previous research, we can observe some subjective symptoms and measure objective changes in cardiovascular values to reflect the susceptibility of motion sickness. The well-known “Graybiel diagnostic criteria” and “vestibular function scale” used by our group showed that the adverse symptoms after three kinds of training on the last day test were lower than the first day of the rotary chair test. In particular, the “Graybiel diagnostic criteria” are more detailed, and can clearly distinguish changes between the three different training methods.

The stress response is known to involve the autonomic nervous system (ANS), including the sympathetic nervous system (SNS) and the parasympathetic nervous system (PNS). It is distributed in human cardiovascular, glandular, and visceral organs and can regulate the activities of the viscera, vascular smooth muscles, myocardium, and glands (Goldberger et al., 2001; O’Connor et al., 2021). Based on previous studies, it has been confirmed that motion sickness caused by exercise can increase sympathetic nerve activity, regulate blood flow distribution and maintain stable cardiovascular function (Barak et al., 2010; Yang et al., 2021). HRV is an important indicator for judging the dynamic balance between the sympathetic nerve and parasympathetic nerve, which can reflect the regulation of the autonomic nervous system on the cardiovascular system (Goncalves et al., 2022). In this study, our results showed that MS induced by the rotary chair leads to an increase in LFn and a decrease in HFn and therefore to a significant increasement in LF/HF, which is consistent with previous research results (Zhao et al., 2022). After hypoxia acclimatization training, we found that LFn decreased and HFn increased, and therefore, this led to a significant decrease in LF/HF in MS induced by the rotary chair, which is consistent with the 3D roller training group and combined 3D roller and hypoxia acclimatization training group. These results suggested that sympathetic activity was increased, vagal activity was decreased in rotary chair-induced MS, and hypoxia acclimatization training improved MS-induced autonomic response of HRV.

The PNS and SNS are responsible for pupil constriction and dilation, respectively. The pupillary response can therefore provide a sensitive, inexpensive, and non-invasive measure that reflects the ANS pathways. The response has been utilized to assess diseases, various cognitive tasks, and autonomic activation (Reddy et al., 2018; Zhou X. et al., 2022). By comparing the results of the participants’ pupillary light reflex in MS induced by the rotary chair and before, we found that the PCV and PMD in both of the participants’ eyes were significantly reduced, which may be related to the changes in the autonomic nervous function. However, the reduction in PCV and PMD was significantly lower in the hypoxia acclimatization training group, the 3D roller training group, and the combined training group in MS induced by the rotary chair. In particular, binocular PCV was further reduced in the combined hypoxia acclimatization and 3D roller training group. Although the present study cannot clearly analyze the effect of pupillary light reflex on MS study, it can be inferred that the change in PCV and PMD may be related to the changes in the autonomic nervous function induced by MS stimulation. Therefore, we can assume that the smaller the change of PCV and PMD, the less the received MS stimulation and the higher the resistance to MS. In this study, we also examined participants’ PID and PCL in both eyes. The results showed that both PID and PCL were reduced in MS induced by the rotary chair (data not shown), but there was no significant difference in changes of PID and PCL between the control and the other three training groups (data not shown). These results suggest that neither hypoxia acclimatization training nor 3D roller training alone, or a combination of the two, changes the PID and PCL in MS induced by the rotary chair.

There are some limitations in this study. One is the relatively high standard deviation of some of the data, which is mainly related to individual differences between subjects. Although we have already assigned 12 participants to each group, the study may need higher numbers. Second, HRV and PLR have been discussed controversially on directly reflecting the functional level of sympathetic and parasympathetic nerves. Third, some data did not differ significantly between the training and control groups, such as changes in PID and PCL. In addition, although data from the PCV-L and PCV-R were measured simultaneously, the difference between the left and right eye was mainly related to the experimental tool. Therefore, the precision of the measurement should be studied and improved in further research.

## 5 Conclusion

In summary, our results demonstrate that both hypoxia acclimatization training and 3D roller training had a similar effect of reducing adverse symptoms after MS was induced by the rotary chair and improving the resistance to MS. Importantly, if the two training methods are combined, the improvement will be more effective. Our research provides a possible candidate solution for training military pilots in MS problems and lays a theoretical foundation for preventing vestibular provocation and ensuring flight safety. Our study also provides potential training options for other occupational groups such as gymnasts for improving the resistance to MS and for treating patients with dizziness.

## Data availability statement

The original contributions presented in the study are included in the article/supplementary material, further inquiries can be directed to the corresponding authors.

## Ethics statement

The studies involving humans were approved by the Medical Ethics Committee of Fourth Military Medical University. The studies were conducted in accordance with the local legislation and institutional requirements. The participants provided their written informed consent to participate in this study.

## Author contributions

XZ and TZ: conceptualization, project administration, and funding acquisition. XZ, RW, YY, and YT: writing—original draft. RW, YY, and YT: methodology, software, validation, formal analysis, and investigation. QZ, YP, SL, JF, CL, XL, YW, and XS: resources. RW and XZ: data curation. XZ, RW, and TZ: writing—reviewing and editing. XZ: visualization and supervision. All authors contributed to the article and approved the submitted version.

## Glossary

**Table:** 

3DRT	3D roller training
ANS	Autonomic nervous system
HAT	Hypoxia acclimatization training
HF	High frequency
HFn	Normalized high frequency
HPC	Hypoxia preconditioning
HRV	Heart rate variability
LF	Low frequency
LFn	Normalized low frequency
MS	Motion sickness
OSAS	Obstructive sleep apnea syndrome
PCL	Pupil contraction latency
PCV	Pupil contraction velocity
PID	Pupil initial diameter
PMD	Pupil minimum diameter
PNS	Parasympathetic nervous system
SD	Standard deviation
SNS	Sympathetic nervous system
